# Effect of pomiferin administration on kidney ischaemia-reperfusion injury in rats

**DOI:** 10.2478/v10102-010-0015-1

**Published:** 2010-06

**Authors:** Lenka Bartošíková, Jiří Nečas, Tomáš Bartošík, Martin Pavlík, Petr Fráňa

**Affiliations:** 1 Department of Physiology, Faculty of Medicine and Dentistry, Palacky University Olomouc, Czech Republic; 2 Department of Anaesthesiology and Intensive Care, St. Anne's University Hospital Brno, Czech Republic; 3 2^nd^ Department of Internal Medicine, St. Anne's University Hospital Brno, Czech Republic

**Keywords:** antioxidative effect, ischaemia-reperfusion, kidney, pomiferin, therapy

## Abstract

The aim of the study was to analyse protective effects of different doses of pomiferin in therapy of reperfusion injury. Rats were randomly divided into five groups (n=10). One group was intact. Three medicated groups and one placebo group were subjected to ischaemia and reperfusion of the left kidney. Pomiferin was administrated by single gastric gavage in 2 ml of 0.5% Avicel solution in doses of 5, 10 and 20 mg/kg. The placebo group was given only Avicel solution. On day 15, all the animals were exsanguinated and the reperfused kidneys were recovered. Selected biochemical markers were assessed in blood: antioxidative enzymes, total antioxidative capacity, malondialdehyde, creatinine, urea and uric acid. Creatinine, urea and total proteins were analysed in urine and 24-hour diuresis was recorded. The kidney tissue samples were used for histopathological examination.

The results confirmed the expected protective effects of pomiferin. Pomiferin supported defensive reactions of the body against free radicals (increased levels of superoxide dismutase, total antioxidative capacity), decreased lipid peroxidation (decreased malondialdehyde) and contributed to the recovery of kidney functions (creatinine and urea in blood). The best biochemical and histopathological results were achieved after pomiferin administration in the dose of 5 mg/kg.

## Introduction

Reactive oxygen or nitrogen species (ROS/RNS) are constantly formed in the organism during physiological processes or they are derived from external sources and eliminated by the antioxidant defence of the organism (Yu, 1994). There are many sources of free radicals in the metabolic pathways: respiration chain in mitochondria, metabolism of catecholamines and prostanoids, xanthine oxidase, NAD(P)H-oxidase, and several other enzymatic systems. Under physiological conditions, a balance exists between the level of ROS and the level of endogenous antioxidants. When this balance is disrupted, oxidative stress occurs. ROS modify cell membrane lipids, proteins and nucleic acids, leading to changes in membrane fluidity and permeability, fragmentation or random cross linking of molecules like DNA, enzymes and structural proteins (Carattino *et al*., 1999; Ratnam *et al*., 2006).

Ischaemia and hypoxia have many biological effects, including depletion of ATP, increased levels of calcium, and alteration in membrane lipids and enzyme activities (Tamura *et al*., 1996). Xanthine dehydrogenase is converted to xanthine oxidase (XOD) during ischaemia. In reperfusion – reoxygenation of ischaemic tissue – XOD becomes the main source of ROS. Cytochrome *c* and apoptosis-inducing factor are released into the cytoplasm (Zhao, 2004). Production of ROS is very fast – in the ischaemic myocardium ROS are detected in the first minutes after reperfusion (Zhao, 2004). ROS attack cells, induce synthesis of adhesive molecules which attract neutrophils – producers of other ROS molecules (Drabikova *et al*., 2002). All these actions cause oxidative injury.

ROS play an important role in the pathogenesis of ischaemia-reperfusion (I/R) injury in the kidney (Serteser *et al*., 2002; Singh & Chopra, 2004). Renal ischaemia is an important clinical problem in renal transplantation, in the early allograft rejection subsequent to renal transplantation, cardiovascular surgery, *etc*. I/R injury can result in acute renal failure. Acute renal failure is a clinical and experimental syndrome characterised by major reduction in glomerular filtration rate, extensive tubular damage, tubular cell necrosis, glomerular injury, and signs of tubular obstruction with cell debris (Singh & Chopra, 2004).

Therapy of ischaemic injury is complicated. The use of preventive approaches – strengthening of natural antioxidative mechanisms – has proved effective (Janostikova *et al*., 2005; Pecivova *et al*., 2004). If this is not possible, I/R injury has to be reduced by therapeutic administration of compounds which act as free radical scavengers.

Flavonoids are a heterogeneous group of phenol compounds. In human diet, the most abundant of them are polyphenols. Many positive and also negative effects of flavonoids have been described (Van Hoorn *et al*., 2002).

Depending on their chemical structure, flavonoids can act as antioxidants and scavengers via several mechanisms (Shon *et al*., 2004). Flavonoids have their own antioxidative capacity and they are able to regulate enzymes such as xanthine oxidase, phospholipase and nitric oxide synthase. They can inhibit peroxynitrite in activated macrophages (Van Hoorn *et al*., 2002).

**Figure 1:**
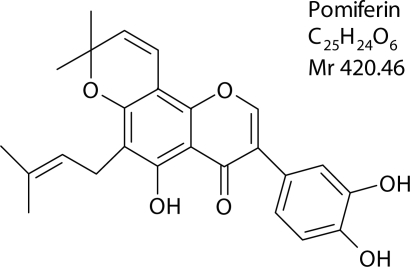
Chemical structure of pomiferin.

Pomiferin 3-(3,4-dihydroxy-phenyl)-5-hydroxy-8,8-dimethyl-6-(3-methyl-but-2-enyl)-8H-pyrano[2,3-f]chromen-4-one (Figure 1) belongs to the group of prenylated isoflavones. The compound was isolated from *Maclura pomifera* (Raf.) Schneid. (Moraceae) and its purity was HPLC-proved (Janostikova *et al*., 2005).

The aim of the study was to analyse the effects of different doses of pomiferin administered during the therapy of ischaemia-reperfusion injury of kidney tissue in laboratory rats.

The study and its experimental protocol were approved and monitored by the Ethical Committee of the University of Veterinary and Pharmaceutical Sciences Brno. The experiment was performed by the work group whose members are holders of the Certificate on Professional Competence issued by the Central Commission for Animal Protection pursuant to §17 of the Act on Protection of Animals against Cruelty (No. 246/1992 Coll.) of the Czech National Council.

## Materials and methods

Male Wistar SPF laboratory rats (origin – AnLab, Germany) were used in the study. All animals were of the same age and comparable weight (250 ± 10 g). They were housed in a room with standard temperature and light regime, fed a standard diet (Complete diet for laboratory mice and rats in SPF breedings – M1; origin – Ing. F. Nachal, VKS Jirikov, Czech Republic, Reg. No. 10250) and received water ad libitum.

After 10 days of acclimation, 50 animals were randomised in 5 groups (n=10). One group was left intact. Four groups underwent laparotomy in general anaesthesia (2% Rometar (xylazine) 0.5 ml + Narkamon (ketamine) 10 ml, dose 0.5 ml solution /100 g of body mass). Ischaemia-reperfusion injury was induced by applying a vascular clamp on the left renal artery for 60 min with subsequent renal reperfusion. Each animal (including those of the intact group) was put into its own metabolic cage. For 15 days all the animals were bred in these cages. The doses of pomiferin were suspended in 2 ml of 0.5% Avicel solution (microcrystalline cellulose) and were administered starting with day 1 after operation. Three of four operated groups were medicated with pomiferin – orally by gastric gavage once a day in different doses: 5 mg/kg, 10 mg/kg and 20 mg/kg. The placebo group was given 2 ml of 0.5% Avicel by the same route. On day 15, all the animals were exsanguinated in general anaesthesia (2% Rometar 0.5 ml + Narkamon 10 ml, dose 0.5 ml solution /100 g of the rat body mass) by blood collection from the left ventricle and the reperfused kidney tissue was employed for histopathological examination.

Selected biochemical markers were assessed in blood: superoxide dismutase (SOD, EC 1.15.1.1) (Arthur and Boyne, 1993), glutathione peroxidase (GSHpx, EC 1.11.1.9) (Paglia and Valentine, 1967), total antioxidative capacity (AOC) (Miller *et al*., 1993) using RANDOX testing kits (Dublin, Ireland), in COBAS MIRA S automatic analyser, and malondialdehyde (MDA) was analysed spectrophotometrically using the TBARs method (Kosugi and Kikugawa, 1989); creatinine, urea and uric acid were assessed in blood by standard laboratory methods using BIO-LA-TEST testing kits (Pliva-Lachema, Czech Republic). Creatinine, urea and total protein were analysed in urine by standard laboratory methods using BIO-LA-TEST testing kits (Pliva-Lachema, Czech Republic). Diuresis/24 hours was measured using a calibrated glass container.

The kidney tissue samples were fixed in 10% formaldehyde and processed manually. Two blocks were made of each sample, the sections were stained with haematoxylin-eosin. All evaluated samples were of outstanding quality. The evaluation was performed by a histopathologist without knowledge of the experimental protocol.

Evaluation principle: each sample of the material was evaluated and scored separately in 3 kidney tissue structures, the results were added up and in the end the average score of each medicated group was stated.

### Scoring schedule

1^st^ tissue structure – kidney medulla – the grade of tissue destruction through bleeding (according to the extent) and presence of inflammatory infiltrate were evaluated, indicated as plus (+) sign (max. 3+)

2^nd^ tissue structure – cortex and glomerules – both extraglomerular (max. 1+) presence of haemorrhages and increased cellularity and extravasates in the glomerule (max. 2++) were evaluated.

3^rd^ tissue structure – kidney tubules – presence of regressive changes of epithelia from edema to necrosis were evaluated (+ in the case of necrosis and ± in the case of regression not reaching the grade of necrosis). In addition, the tubule content was evaluated (protein and hyaline cylinders +). Maximum 2++.

The most extensive damage – the highest possible score per one sample was 7.

### Statistical analysis

The results were processed by the Microsoft^®^ Excel^®^ table processor and statistically interpreted using Unistat programme and ANOVA test. The value *p*≤0.05 was considered significant.

## Results

All the results were obtained from samples (blood, urine, kidney tissue) of the 15^th^ day of the experiment.

### Results of biochemical examination

Biochemical results are given in Tables 1–3. A statistically highly significant increase of SOD activities (*p*≤0.01) was detected in the groups medicated with the doses of 5, 10, and 20 mg/kg, compared with the placebo and the intact group. Mutual comparison of the SOD activities among the groups medicated with different doses of pomiferin did not show significant differences and the difference between the placebo and the intact group was statistically nonsignificant.

**Table 1:** Biochemical markers of I/R injury assessed in blood, expressed as X ± SD.

Group of animals (n=10)		SOD (U/ml)	GSHPx (μkat/L)	AOC (mmol/L)	MDA (mmol/L)
**Medicated groups** Dose of pomiferin	5 mg/kg	234.45 ± 3.54 **++	1036.58 ± 52.28 **++	0.57 ± 0.02 **++	0.40 ± 0.30 **++
	10 mg/kg	240.03 ± 17.56 **++	1138.50 ± 67.28 **++	0.53 ± 0.07 **++	4.11 ± 1.42 **++
	20 mg/kg	234.54 ± 16.79 **++	1064.75 ± 53.25 **++	0.58 ± 0.05 **++	4.23 ± 1.11 **++
Placebo group		70.39 ± 2.79	1329.00 ± 91.41 ‡‡	0.41 ± 0.04	17.17 ± 1.12 ‡‡
**Intact group**		67.37 ± 3.97	1509.38 ± 147.93	0.43 ± 0.03	1.89 ± 0.16

^*^ *p*≤0.05 treated vs placebo group;

^**^ *p*≤0.01 tretaed vs placebo group

+ *p*≤0.05 treated vs intact group;

++ *p*≤0.01 treated vs intact group

‡ *p*≤0.05 placebo vs intact group;

‡‡ *p*≤0.01 placebo vs intact group

**Table 2:** Biochemical markers of kidney function assessed in blood, expressed as X ± SD.

Group of animals (n=10)		Creatinine (μmol/ml)	Urea (mmol/L)	Uric Acid (μmol/ml)
**Medicated groups** Dose of pomiferin	5 mg/kg	43.43 ± 4.72	6.57 ± 1.51 +	22.05 ± 6.82
	10 mg/kg	44.60 ± 1.95+	7.61 ± 1.04 ++	16.00 ± 3.36
	20 mg/kg	37.91 ± 2.50 **++	6.72 ± 1.00	34.45 ± 15.71 *+
**Placebo group**		44.83 ± 1.78 ‡‡	7.21 ± 0.56 ‡‡	18.91 ± 5.64
**Intact group**		42.00 ± 1.94	6.35 ± 0.25	18.68 ± 4.00

^*^ *p*≤0.05 treated vs placebo group;

^**^ *p*≤0.01 tretaed vs placebo group

+ *p*≤0.05 treated vs intact group;

++ *p*≤0.01 treated vs intact group

‡*p*≤0.05 placebo vs intact group;

‡‡ *p*≤0.01 placebo vs intact group

**Table 3:** Biochemical markers of kidney function assessed in urine, expressed as X ± SD.

Group of animals (n=10)		Creatinine (μmol/ml)	Urea (mmol/L)	Total Protein (g/L)	Diuresis/24 h (ml)
**Medicated groups** Dose of pomiferin	5 mg/kg	1731.45 ± 433.95 *+	289.23 ± 73.07 ++	1.16 ± 0.21 *++	25.75 ± 5.47 ++
	10 mg/kg	2232.60 ± 960.90 +	378.33 ± 219.60 +	1.34 ± 0.36 *++	28.89 ± 12.10 ++
	20 mg/kg	1795.35 ± 492.93 *+	322.73 ± 129.18 ++	1.36 ± 0.35 **++	31.19 ± 9.11 *++
**Placebo group**		3245.90 ± 1392.93 ‡‡	426.01 ± 241.78 ‡	0.83 ± 0.37 ‡	19.63 ± 10.75 ‡‡
**Intact group**		1152.21 ± 453.88	143.88 ± 67.25	0.43 ± 0.16	50.16 ± 10.44

^*^ *p*≤0.05 treated vs placebo group;

^**^ *p*≤0.01 tretaed vs placebo group

+ *p*≤0.05 treated vs intact group;

++ *p*≤0.01 treated vs intact group

‡ *p*≤0.05 placebo vs intact group;

‡‡ *p*≤0.01 placebo vs intact group

GSHPx activities in all medicated groups were significantly decreased (*p*≤0.01), compared with the placebo and the intact group. Comparison of GSHPx activities ascertained in the groups medicated with doses of 5 and 10 mg/kg showed a significant difference (*p*≤0.01), as did the comparison between the groups medicated with doses of 10 and 20 mg/kg (*p*≤0.05). GSHPx activity in the placebo group was decreased (*p*≤0.01) compared with the intact group.

A significant increase of AOC values (*p*≤0.01) was detected in all groups medicated with pomiferin, compared with the placebo and the intact group but mutual comparison among all medicated groups as well as between the placebo and the intact group were nonsignificant.

The MDA concentrations in blood were significantly (*p*≤0.01) decreased in all medicated groups compared with the placebo group. Compared with the intact group, MDA concentration was decreased in the group medicated with the doses of 5 mg/kg and increased in the groups medicated with pomiferin in the doses of 10 and 20 mg/kg. Comparison of the concentrations ascertained in the placebo and intact group showed an increase (*p*≤0.01) of MDA concentration in the placebo group.

The creatinine concentration was significantly decreased (*p*≤0.01) in the group medicated with the dose of 20 mg/kg, compared with the placebo and the intact group. A significantly increased concentraion of creatinine (*p*≤0.05) was detected in the group medicated with the dose of 10 mg/kg, compared with the intact group. In the placebo group, the creatinine concentration was significantly increased (*p*≤0.01) compared with the intact group. The creatinine concentration closest to the level of the intact group was detected in the group medicated with pomiferin in the dose of 5 mg/kg. In the mutual comparison, significant differences were recorded between the groups medicated with the doses of 5 and 20 mg/kg (*p*≤0.05) and between the groups medicated with the doses of 10 and 20 mg/kg (*p*≤0.01). The creatinine concentration was increased (*p*≤0.01) in the placebo group, compared with the intact group.

The urea concentrations were increased (*p*≤0.01) in the group medicated with the dose of 10 mg/kg and the placebo group, compared with the intact group. The urea concentration closest to the level of the intact group was detected in the group treated with the dose of 5 mg/kg. Mutual comparison of the urea concentrations among the groups medicated with different doses of pomiferin did not show significant differences. Comparison of the values obtained in the placebo group and the intact group showed an increase (*p*≤0.01) of urea concentration in the placebo group.

The concentration of uric acid was increased (*p*≤0.05) in the group medicated with the dose of 20 mg/kg, compared with the placebo and the intact group. The uric acid value closest to the concentration of the intact group was detected in the group medicated with the dose of 10 mg/kg. Comparison of uric acid concentrations obtained in the groups medicated with pomiferin in doses of 5 and 10 mg/kg, 5 and 20 mg/kg, and 10 and 20 mg/kg showed significant differences (*p*≤0.05). Mutual comparison of uric acid concentrations in all medicated groups showed significant differences (*p*≤0.05). There was no significant difference between the placebo and intact animal group.

A significant increase of creatinine concentration (*p*≤0.05) was detected in all groups medicated with pomiferin, compared with the intact group and a decrease (*p*≤0.05) in the groups medicated with the dose of 5 and 20 mg/kg, compared with the placebo group. The creatinine concentration closest to the concentration of the intact group was detected in the group medicated with the dose of 5 mg/kg. Mutual comparison of creatinine concentrations in all medicated groups did not show significant differences. The creatinine concentration was increased (*p*≤0.01) in the placebo group, compared with the intact group.

Urea concentrations were increased (*p*≤0.01 and *p*≤0.05) in the groups medicated with the respective doses of 5 and 20 mg/kg and 5 and 10 mg/kg, compared with the intact group. The urea concentration closest to the concentration of the intact group was detected in the group medicated with the dose of 5 mg/kg. Mutual comparisons of urea concentrations obtained in all medicated groups were nonsignificant. The urea concentration was increased (*p*≤0.05) in the placebo group, compared with the intact group.

The total protein concentration was increased (*p*≤0.01) in all medicated groups, compared with the intact group. The total protein concentration was increased in the groups medicated with the dose of 5 and 10 mg/kg (*p*≤0.05) and in the group medicated with the dose of 20 mg/kg (*p*≤0.01), compared with the placebo group. The total protein concentration closest to the concentration of the intact group was detected in the group medicated with the dose of 5 mg/kg. Mutual comparisons of total protein concentrations recorded in all medicated groups were nonsignificant. The total protein concentration was increased (*p*≤0.05) in the placebo group, compared with the intact group.

A significant decrease of daily diuresis (*p*≤0.01) was detected in all groups medicated with pomiferin and in the placebo group, compared with the intact group. The daily diuresis closest to the value of the intact group was detected in the group medicated with the dose of 20 mg/kg. Mutual comparisons of daily diuresis as measured in all medicated groups were nonsignificant. The daily diuresis value in the placebo group was decreased (*p*≤0.01), compared with the intact group.

### Results of histopathological examination

#### The medicated groups

The optimal protective effect appeared after administration of pomiferin in the dose of 5 mg/kg. The average score of the samples of this group was 3.3. In the 1^st^ tissue structure (kidney medulla) the best result was achieved after administration of the highest dose of pomiferin – 20 mg/kg, the average score of this tissue structure being 1.0. In all medicated groups, the average presence of the inflammation was 88%.

#### The placebo group

Typical ischaemic changes were observed in all samples. The score was oscillating from 2.0 to 5.5 with the average of 4.3. Inflammatory infiltrate was found rather frequently along with the presence of polymorphonuclear neutrophils (67%).

#### The intact control group

Haemorrhage occurred only accidentally, most probably caused by contusion.

## DISCUSSION

The prenylated isoflavonoid pomiferin is a substance which was only rarely tested *in vivo* (Janostikova *et al*., 2005; Florian *et al*., 2006). Its antioxidative effect may be achieved by several mechanisms. According to its structure, it could chelate copper and iron, which are potential inducers of Fenton reaction and it could also inactivate xanthine oxidase (Ratnam *et al*., 2006). Generally, flavonoids are able to inactivate hydrogen peroxide, peroxyl radicals, and thus protect lipids against peroxidation, inhibit activation of phospholipase A_2_ and degradation of arachidonic acid in lipid membranes (Bors *et al*., 1990).

Our study analysed the relation between the therapeutic effect and the dose of pomiferin used under the conditions of kidney ischaemia-reperfusion in the laboratory rat.

The statistically significant higher SOD activities found in the medicated groups confirmed the readiness of the organism to destroy superoxide. In our study, the activity of SOD in the treated groups was approximately three times higher than in the intact group. In a similar type of study (Singh and Chopra, 2004) the tested flavonoid naringin decreased the activity of SOD to 50% compared to sham control. There was no linear relation between the dose of pomiferin and the activity of SOD in our study. The highest effect on SOD activity increase was found with the middle dose – 10 mg/kg.

**Table 4:** Final scoring for all treated groups.

						in blood			in urine					
								
Group of animals (n=10)		SOD	GSHPx	AOC	MDA	Creatinine	Urea	Uric acid	Creatinine	Urea	Total protein	Diuresis/24 h	Histopathology	Final evaluation
	5 mg/kg				•	•	•		•	•	•		•	7
**Dose of pomiferin**	10 mg/kg	•	•					•						3
	20 mg/kg			•								•		2

• indicates the best result for each evaluated parameter or biochemical marker

Statistically significant was the decrease of GSHPx activities in all medicated groups. With the middle dose – 10 mg/kg – the GSHPx activity was the highest.

Since the analysed enzymes are intracellular and their activities follow one another, we can suppose that their concentrations are influenced by the type of ROS that is actually present. The massive production of SOD may have been a physiological response, supported by the effect of pomiferin, to the presence of the huge amount of superoxide, whereas GSHPx did not have hydrogen peroxide enough at the moment of assessment, and thus its activities were not increased yet. Pomiferin could also co-operate in scavenging actions and this way influence the level of irritating type of ROS. The detailed mechanisms of these supposed actions are unknown. The views on changes of SOD and GSHPx activities caused by declining kidney function are inconsistent – some authors suggest increased activities (Chen *et al*., 1996), others decreased (Racek *et al*., 1995, Singh & Chopra, 2004), or unaffected activities (Durackova, 1997).

AOC is not the most specific determinant of antioxidative abilities of the organism because it is a sum of effects of many antioxidative factors. Uric acid, which is the final product of degradation of AMP, is a very important antioxidant in blood. In our study, uric acid was significantly (*p*≤0.05) increased only in one case – after the administration of pomiferin in the dose of 20 mg/kg, whereas AOC was significantly increased (*p*≤0.01) in all medicated groups. Flavonoids or other compounds with potential antioxidative effect usually increase AOC (Toborek *et al*., 1992; Jackson *et al*., 1995).

MDA is a toxic byproduct of lipoperoxidation and its concentration usually correlates with the intensity of ROS action in lipid membranes and thus also with the severity of oxidative injury. Increased concentration of MDA may be caused by decreased renal elimination during renal failure (Racek *et al*., 1997). Although the TBARs method (Kosugi & Kikugawa, 1989) with spectrophotometrical assessment is not the most specific one, it is sufficient for our measurements, because it is the difference between the assessed concentrations in medicated groups and the placebo or intact group that is important, not the exact quantity of TBARs products. A statistically highly significant decrease of MDA concentrations was recorded in all medicated groups in our study. This result correlates with the assumption of inhibition of lipoperoxidation by flavonoids – in our case pomiferin. The best result was achieved after administration of the lowest dose of pomiferin – 5 mg/kg. This value was even lower than the level of MDA in the intact group.

Acute renal failure was evident particularly in the placebo group. Elevated levels of creatinine and urea in blood and urine along with reduction of daily diuresis are results of homeostatic imbalance caused by renal failure. After fifteen days of pomiferin medication, biochemical markers reached practically physiological levels. In the medicated groups, the positive influence of pomiferin was manifested in daily diuresis, which was higher than in the placebo group.

Histopathological results allow to describe the measure of damage quite exactly. There were mostly inflammatory and necrotic changes it this type of experiment where the reperfusion after 60 minutes of ischaemia lasted 15 days. The placebo group with mostly massive ischaemic changes reached 4.3 points in average. The medicated groups achieved better results, the best one (3.3) was recorded after pomiferin administration in the dose of 5 mg/kg.

Biochemical and histopathological results corresponded and confirmed the antioxidative capacity of pomiferin. The relation between the dose and the effect was linear only in few parameters (MDA, total protein and daily diuresis). The best results in biochemical parameters were reached by the group medicated with pomiferin in the dose of 5 mg/kg and the histopathological examination confirmed this dose as the most effective one. We can conclude that pomiferin medication supported strongly the physiological antioxidative system, contributed to the antioxidative capacity and thus reduced lipoperoxidation of lipids in cell membranes. By all these ways, pomiferin participated in the preservation of vitality of the filtration system in the kidney.

The results of this study can be used to advantage in further preclinical examinations of pomiferin.
